# Polystyrene Microplastics Exacerbate *Candida albicans* Infection Ability In Vitro and In Vivo

**DOI:** 10.3390/ijms25010012

**Published:** 2023-12-19

**Authors:** Angela Maione, Mariangela Norcia, Marica Sinoca, Marilena Galdiero, Valeria Maselli, Antonia Feola, Rosa Carotenuto, Paola Cuomo, Rosanna Capparelli, Marco Guida, Emilia Galdiero

**Affiliations:** 1 Department of Biology, University of Naples Federico II, Via Cinthia, 80126 Naples, Italy; angela.maione@unina.it (A.M.); mariangela.norcia@unina.it (M.N.); marica.sinoca@unina.it (M.S.); valeria.maselli@unina.it (V.M.); antonia.feola@unina.it (A.F.); rosa.carotenuto@unina.it (R.C.); 2 Department of Experimental Medicine, University of Campania “Luigi Vanvitelli”, 81100 Naples, Italy; marilena.galdiero@unicampania.it; 3 Department of Agricultural Sciences, University of Naples Federico II, 80055 Portici, Italy; paola.cuomo@unina.it (P.C.); rosanna.capparelli@unina.it (R.C.); 4 National Biodiversity Future Center (NBFC), 90133 Palermo, Italy; 5 Center for Studies on Bioinspired Agro-Environmental Technology (BAT Center), 80055 Portici, Italy

**Keywords:** polystyrene microplastics, *Candida albicans*, *Galleria mellonella*, biofilm, cytokines, toxicity

## Abstract

Plastic pollution is an important environmental problem, and microplastics have been shown to have harmful effects on human and animal health, affecting immune and metabolic physiological functions. Further, microplastics can interfere with commensal microorganisms and exert deleterious effects on exposure to pathogens. Here, we compared the effects of 1 µm diameter polystyrene microplastic (PSMPs) on *Candida albicans* infection in both in vitro and in vivo models by using HT29 cells and *Galleria mellonella* larvae, respectively. The results demonstrated that PSMPs could promote *Candida* infection in HT29 cells and larvae of *G. mellonella*, which show immune responses similar to vertebrates. In this study, we provide new experimental evidence for the risk to human health posed by PSMPs in conjunction with *Candida* infections.

## 1. Introduction

Plastic wastes are environmentally ubiquitous and consequently microplastics (MPs) pollution has become a great concern worldwide [1]. MPs are plastic fragments smaller than 5 mm in size that can derive from plastic fragments by photodegradation, oxidation, hydrolytic and mechanical degradation. Some MPs are also deliberately used as components in some cosmetics and personal hygiene products. Even if most studies are focused on MPs occurrence and their effects in marine environment [2], more than in soil and airborne environment [3], ingestion is considered one of the main routes for potential human exposure to MPs, through contaminated food and liquids. Indeed, MPs can be ingested by eating contaminated seafood, sugar, salt, beer, and tap and bottled water, as well as through food packaging (plastic coffee capsules) [4].

MPs can reach a variety of organisms through food chains and can accumulate in the human body, including in blood, sputum, pregnant embryos, and lungs; their accumulation in the lungs mainly results from the inhalation of MP-containing aerosols [5]. In addition to their own possible toxicity, MPs’ derivatives, such as phthalates and bisphenol A, are known as endocrine disruptors which interfere with endogenous hormones [6] even at very low concentrations.

Despite the fact that the hazard for human exposure to ingested MPs is potentially high, maybe leading to a chronic inflammatory condition, experimental data on the effects of this type of exposure are very limited [7], and the risk to the human gastrointestinal tract remains elusive. Numerous in vivo and in vitro experiments have demonstrated that MPs could cause oxidative stress; induce inflammatory responses, focusing specifically on the activation of macrophages and the production of related cytokine; disrupt epithelial barrier function; and modulate intestinal microbiota and immune homeostasis [8]. Furthermore, MPs can function as vectors of pathogen microorganisms that can colonize their surface, transporting microorganisms into tissues, protecting them from the host immune system, and creating tissue damage that can favor infection [9]. Current evidence indicates that MPs could be colonized by microorganisms also organized in biofilms that act as reservoirs for pathogenic bacteria, and they especially are involved in the horizontal gene transfer that regulates the flow of antibiotic resistance genes (ARGs) between the sessile microorganisms in the biofilm and the environmental bacteria [10,11].

Fungi are among the most prevalent opportunistic pathogens confirmed as one of the threats to human life. The human fungal pathogen *Candida albicans* is frequently found in the oral cavity, the genital area, and also the gastrointestinal tract of many individuals [12]. The effects of the occurrence of MPs in the gastrointestinal tract in concomitance with *Candida* infection are still unclear. To the best of our knowledge, there are no reports on the interactions between MPs and *Candida* in intestinal cell populations, and on the invasive ability and infection capacity of the fungus in such cell populations upon MPs exposure.

Therefore, the aim of this study was to establish whether the presence of polystyrene microplastics (PSMPs) could interfere with the ability of *C. albicans* cells to colonize the host both in vitro and in vivo.

Polystyrene is a thermoplastic polymer which is widely used in disposable drinking cups, food packaging, electronic products, and personal care products [13]. In order to assess the potential risk for human health derived from PSMPs combined with the yeast *C. albicans*, two sets of experiments were designed. The first was conducted on the human intestinal epithelial cell line HT29 with the purpose of evaluating if different conditions of exposure to PSMPs could promote the invasion and infection ability of a *C. albicans* clinical isolate and improve cell injury and inflammation. The second set of experiments was conducted in vivo, using *Galleria mellonella* larvae as a model to evaluate not only larvae survival in different conditions of PSMPs exposure but also their innate immune response.

## 2. Results

### 2.1. Characteristics of PSMPs

**Figure 1:**
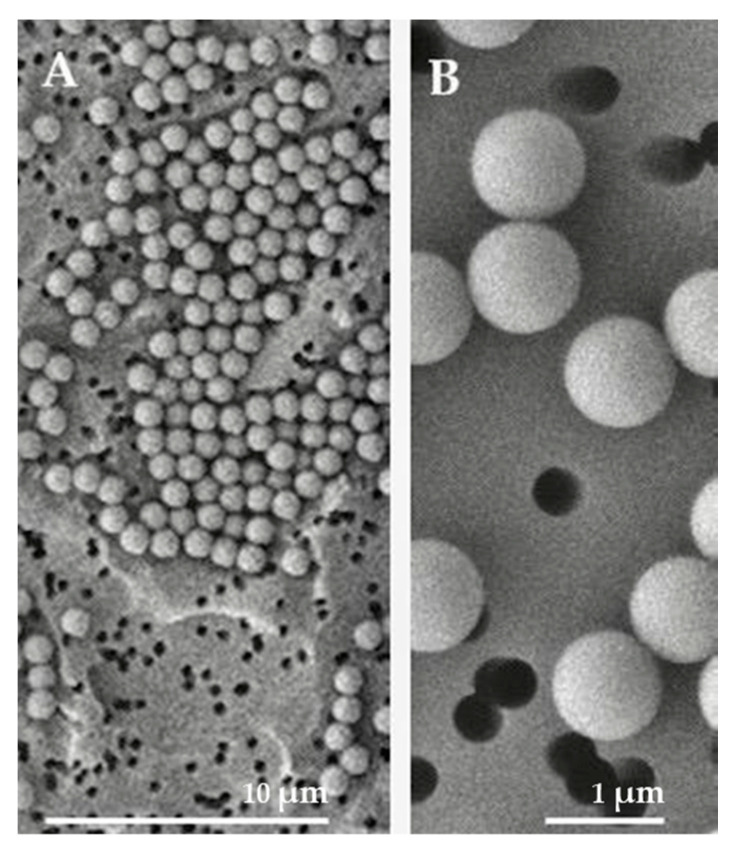
SEM microscopy visualization of PSMPs at two different magnifications ((**A**) 1000× and (**B**) 5000×). PSMPs showed a spherical shape and a uniform diameter of 1 µm.

Photographs obtained under a scanning electron microscope (Figure 1) showed that the microplastics used, in accordance with the data provided by the manufacturing company, are uniformly spherical and have a diameter of 1 micron.

### 2.2. Toxic Effect of PSMPs on HT29 Cells

**Figure 2:**
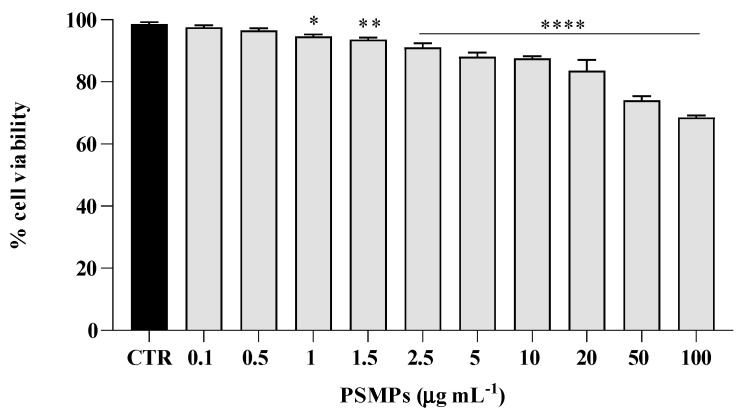
Viability of HT29 cell line challenged by PSMPs shown as the percent cell viability of the cells treated with different concentrations for 24 h. Untreated cells were used as a control. The assays were performed in three independent experiments. One-way ANOVA, followed by Dunnett’s test, was performed to determinate statistically significant results. * = *p* < 0.05, ** = *p* < 0.01, and **** = *p* < 0.0001.

To determine the potential toxicity of polystyrene microplastics on HT29 cells, we evaluated the effect of PSMPs’ (0.1–100 μg mL^−1^) exposure on cell viability. The results, as reported in Figure 2, showed that cell viability decreased at concentrations of PSMPs higher than 2.5 μg mL^−1^, with a decrease of more than 20% only at concentrations of 50 and 100 μg mL^−1^.

### 2.3. FACScan Results

Since it is known that microparticles can be internalized in cancer cells [14], we investigated if internalization and accumulation in HT29 cells after exposure could affect cell morphology. To this end, we analyzed cells via FACScan. The results demonstrated (Supplementary Figure S1) that following the PSMPs’ exposure, the number of viable cells is halved 45.6% ± 2.7 vs. 21.9% ± 2.3 of total events, according to the cell viability test made, but their size did not change, as the measurement of forward scatter, which allows for the discrimination of cell size, did not increase. Interestingly, we noted an increased relative complexity in 40.6% ± 1.4 of cell treated with PSMPs compared to the 3.58% ± 1.2 of the untreated ones, maybe due to PSMPs cell internalization. Moreover, PSMPs not only decreased the total live cell numbers, but the percentage of cells in the G2/M phase of the cell cycle also decreased (5.13% vs. 11.4%), suggesting that PSMPs impaired cell cycle progression.

### 2.4. C. albicans Adheres to PSMPs to Form a Biofilm

**Figure 3:**
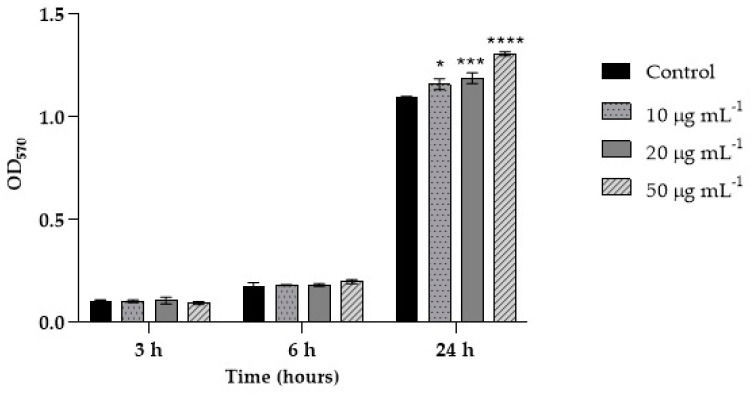
Biofilm formation capacity of C12 during initial attachment (3 h), biofilm formation (6 h), and biofilm maturation (24 h) in the presence or not of different concentrations of PSMPs (10, 20, and 50 μg mL^−1^), using the crystal violet staining method. Results are expressed as the mean of three independent experiments ± standard deviations. One-way ANOVA, followed by Dunnett’s test, was performed to determinate statistically significant results. * = *p* < 0.05, *** = *p* < 0.001, and **** = *p* < 0.0001.

Biofilm formation is one of the main virulence factors of the *C. albicans* clinical isolate C12; therefore, its ability to form a biofilm in the presence of different concentrations of PSMPs was evaluated here. In particular, such an ability was evaluated by analyzing, in vitro, different steps of the biofilm’s development (initial attachment, formation, and maturation), as previously described [15]. As shown in Figure 3, the initial fungal attachment and the second step of biofilm formation were the same for all three conditions and not different from the control. In contrast, after 24 h, when the mature biofilms were evaluated, a significant and higher adhered biomass was detected in the presence of PSMPs, closely linked to their concentration.

### 2.5. Evaluation of C. albicans Invasion Capability

As the human body may be exposed to microplastic pollution at relatively low concentrations, and considering the previous results on toxicity (Figure 2) and biofilm formation (Figure 3), the next experiments were performed using a PSMP concentration of 20 μg mL^−1^. In all three experiments planned, HT29 cells were infected with C12 at MOI 1:5 always for 6 h, while PSMPs were added to cells at different times, i.e., 3, 6, and 24 h.

**Figure 4:**
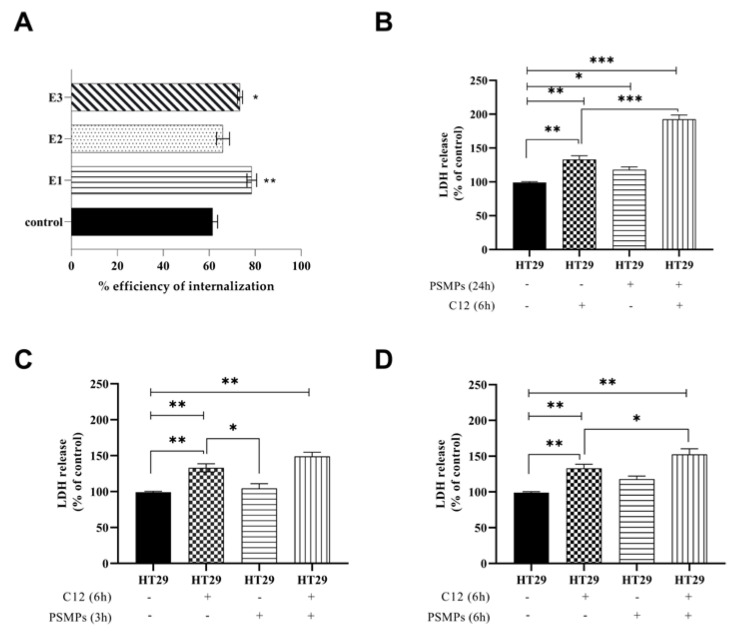
(**A**) Efficiency of internalization of C12 alone (control) and in E1 (PSMPs for 24 h and C12 6 h), E2 (C12 for 6 h and PSMPs for 3 h), and E3 (C12 for 6 h + PSMPs for 6 h). (**B**–**D**) Measurement of LDH release levels in the same conditions E1 (panel (**B**)), E2 (panel (**C**)), and E3 (panel (**D**)). In all panels, HT29 cells untreated (control). Data were obtained from three independent experiments, and the results are presented as the mean ± standard deviation. One-way ANOVA, followed by Dunnett’s test, was performed to determinate statistically significant results. * = *p* < 0.05, ** = *p* <0.01, *** = *p* < 0.001.

To clarify whether PSMPs promote the colonization of *C. albicans* in HT29 cell line (E1), to detect whether a previous infection with *C. albicans* improves cell damages due to PSMPs (E2), and to study the synergistic effects of PSMPs and *C. albicans* on cell injury (E3), we evaluated the percentage of *Candida* internalization in the three conditions and, in parallel, the cell damage in terms of the release of lactate dehydrogenase (LDH) in the cell supernatants (Figure 4).

The results, as shown in Figure 4A, showed that the efficiency of internalization of *C. albicans* in E1 was about 80%, showing an increase in the invasiveness of approximately 20% compared to the infected cells alone. In the case of the co-exposure (E3), the internalization of *Candida* was slightly affected by the presence of PSMPs. Instead, the internalization of C12 was not affected when the PSMPs were added after infection (E2).

Figure 4B–D show the results of cell damage in the three conditions examined. As reported in all panels, the cells infected with C12 for 6 h showed a significant increase in LDH release of 35% (*p* < 0.01) compared to the control (nontreated cells). When cells were treated with PSMPs alone for 3 and 6 h (Panel C and D), we did not observe a significant LDH release; instead, when cells were exposed to PSMPs for 24 h, the release of LDH was significant, *p* < 0.05, with respect to untreated cells (panel B).

Both the conditions of pre-exposure (E1) and co-exposure (E3) (Figure 4B,D) of cells with PSMPs induced an increase in cellular damage of 60% and 30%, respectively, with respect to C12 alone in infected cells (*p* < 0.001 and *p* < 0.05, respectively). On the contrary, post-exposure to PSMPs (E2) did not cause significant cellular damage compared to the C12-infected cells (Figure 4C). Overall, these results indicate that PSMPs can promote fungal infection.

### 2.6. Effect of PSMPs on the Modulation of Inflammatory Cytokine Activity in HT29 Cells

**Figure 5:**
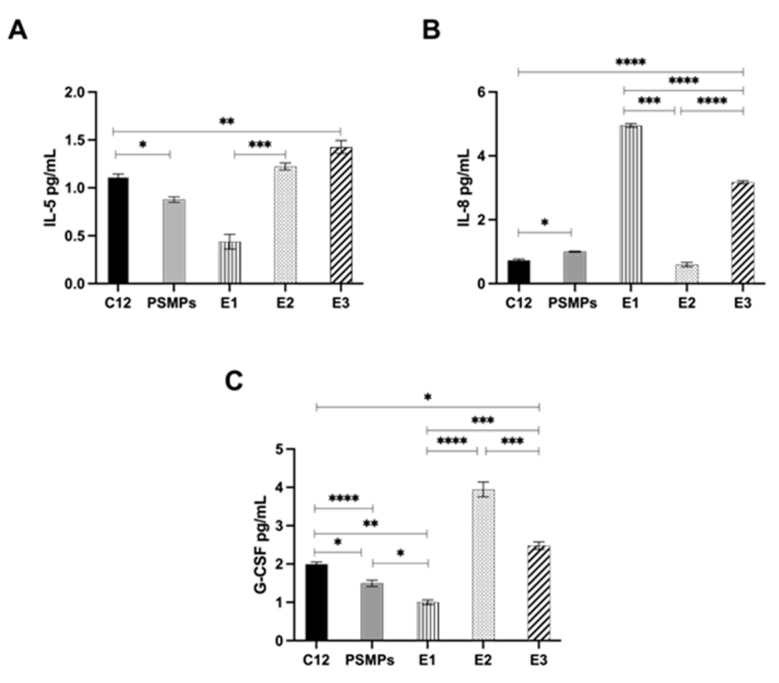
Extracellular levels of IL-5 (**A**), IL-8 (**B**), and G-CSF (**C**) cytokines during C12 infection and exposure to PSMPs: pre-exposure (E1), post-exposure (E2), and co-exposure (E3). Results are normalized to untreated cells (control) and are represented as the means ± standard deviation of two independent experiments, each performed in triplicate. The ordinary one-way ANOVA, followed by Turkey’s post hoc correction, was performed to determinate statistically significant results. * *p* < 0.05, ** *p* < 0.01, *** *p* < 0.001 and ****p < 0.0001.

To further define the role of PSMPs and their immunomodulatory activity, we determined the levels of inflammatory cytokines in all three conditions. In line with previous results (Figure 4), both the pre-exposure and co-exposure of HT29 cells to PSMPs increased the extracellular release of the IL-8 cytokine compared to cells stimulated with C12 alone (Figure 5B). IL-8 is a chemoattractant cytokine that is responsible for the recruitment of polymorphonuclear cells (PMNs) at the site of infection. In addition, it has been reported to occur during gastrointestinal inflammation, promoting tissue damage [16]. Therefore, this result confirms the role of PSMPs as a carrier of the fungal pathogen that is able to enhance the pathogen invasiveness and the related harmful effects. On the contrary, both IL-5 and G-CSF cytokines decreased in cells pre-exposed to PSMPs compared to those co-exposed or exposed to C12 alone (Figure 5A,C). IL-5, as a type-2 immunity cytokine, and the granulocyte colony stimulating factor (G-CSF) play a key role in the resolution of inflammation and tissue repair [17,18], thus justifying their significant decrease in cells pretreated with PSMPs. Taken together, these results indicate that PSMPs can promote fungal infection and support the hypothesis that microplastics could activate late cell apoptosis.

### 2.7. In Vivo Survival Analysis

**Figure 6:**
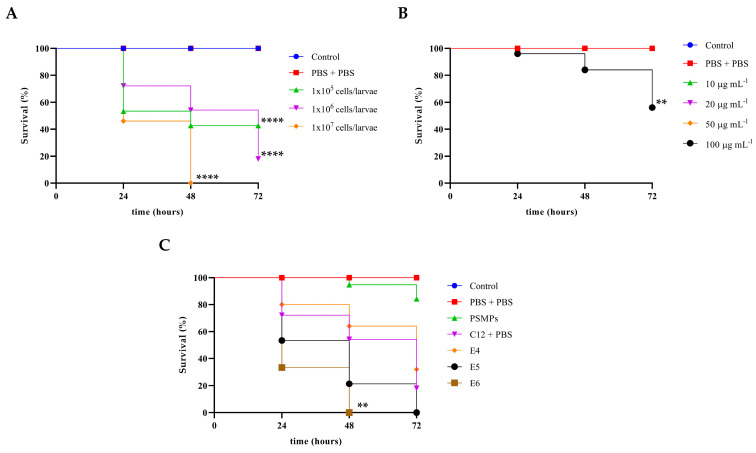
Kaplan–Meier plots of survival curves of *G. mellonella* larvae. (**A**) *G. mellonella* larvae (20 in each group) were infected with serial concentrations of *Candida* cells (from 1 × 10^5^ to 1 × 10^7^ cells/larva). Asterisks represent significant difference vs. PBS + PBS. (**B**) *G. mellonella* larvae (20 in each group) were inoculated with different concentrations of PSMPs (10, 20, 50, and 100 µg mL^−1^). Asterisks represent significant difference vs. PBS + PBS. (**C**) *G. mellonella* larvae (20 in each group) were inoculated with 1 × 10^5^ cells/larva and PSMPs (20 µg mL^−1^) before (E4), after (E5), or simultaneously to (E6) the infection. The control groups were composed of untreated *G. mellonella* larvae (20 in each group) and larvae (20 in each group) that received only a PBS injection. Asterisks represent significant differences. ** = *p* < 0.01, and **** = *p* < 0.0001, log-rank (Mantel–Cox) test.

The toxicity of PSMPs through *G. mellonella* larvae, used in predicting in vivo toxicity, is of fundamental importance, making a connection between in vitro and in vivo assays also because this assay is an alternative in vivo model that is increasingly gaining space due to its advantages, such as its low cost, low biological risk, greater ethical acceptance, and, above all, the similarity of the immune system of these larvae to that of mammals’ innate immune response [19]. In order to identify a suitable inoculum for subsequent experiments, three different *C. albicans* C12 inocula (1 × 10^5^ cells/larva, 1 × 10^6^ cells/larva, and 1 × 10^7^ cells/larva) were each used to infect 20 larvae, and survival was monitored over 72 h. The concentration of of 1 × 10^5^ cells/larva (Figure 6A) was also chosen for to perform the other assays due to larvae survival of about 50% after 48 h.

Three different conditions were carried out to evaluate the effects of PSMPs on *C*. *albicans* infections, as can be seen in Figure 6B. PSMPs did not cause death or toxicity signs in the larvae, suggesting that their use can be considered safe at all doses tested except for the highest one, where the observed survival was 50% in 72 h. The infection with *C. albicans* and PSMPs at concentration of 20 μg mL^−1^ caused survival in 60% of the larvae within 48 h. When the larvae were inoculated with PSMPs prior to *C. albicans* infection or co-administered it, the survival decreased significantly by 20% and completely (i.e., death), respectively (Figure 6C).

### 2.8. Effects of PSMPs on the Expression of the Gene Encoding Gallerimycin and Galiomicin

To investigate the immune mechanisms associated with the effects of PSMPs during *C. albicans* infection, we investigated the gene expression of Galiomicin and Gallerimycin in *G. mellonella*, and we evaluated the persistence of *Candida* virulence by analyzing *HWP1* and *ALS3* genes in the same conditions in larvae.

Galiomicin is a defensin-like peptide that modulates pathogen load and prevents the occurrence of an infection, and Gallerimycin is a cysteine-rich antifungal peptide; both are involved in the immune response to a change in the expression of the gene encoding.

**Figure 7:**
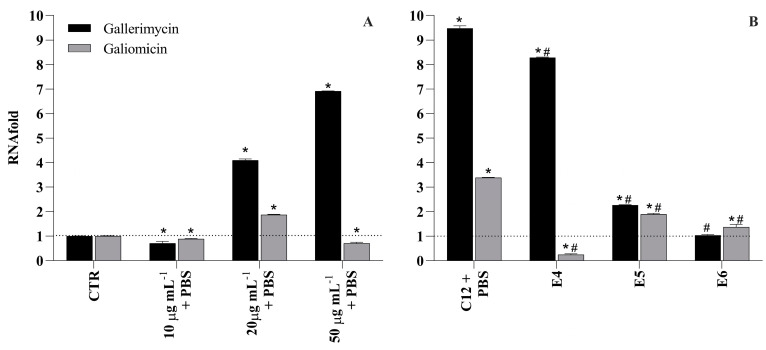
Gene expression analysis for mRNA of Galiomicin and Gallerimycin genes in *G. mellonella* larvae. Relative mRNA expression levels measured using real-time PCR analysis and calculated via the 2^(−∆∆C(T))^ method. Actin gene was used as the housekeeping gene for the normalization of gene expression. (**A**) The three concentrations of microplastics to which the larvae were subjected. (**B**) The larvae infected with C12 only and exposed after 2 h to PSMPs (E4), those that underwent treatment with PSMPs and then C12 (E5), and samples treated simultaneously with PSMPs and C12 (E6). The relative fold change in mRNA in genes expression was compared with that of intact larvae (set y = 1). Each sample was tested and run in duplicate. Error bars represent the SEM. * = *p* < 0.05 with CTR, and # = *p* < 0.05 with C12 + PBS (Wilcoxon two-group test).

To determine the effect of PSMPs on humoral components of insect innate immunity, we first used the RT-PCR analysis to investigate the expression of antifungal peptides in vivo. The Gallerimycin gene expression increases as the concentration of injected microplastics increases, starting from 20 μg mL^−1^ PSMPs and rising 6.9-fold that control at 50 μg mL^−1^ PSMPs. When the injected microplastics were at 10 μg mL^−1^ PSMPs, the Gallerimycin gene did not show any statistical difference from the control (Figure 7A). The second peptide, the Galiomicin gene, is upregulated only in larvae with 20 μg mL^−1^ PSMPs; the other two conditions did not show any statistical difference from the control (Figure 7A).

After 24 h post-inoculation with C12 alone, in comparison to the intact controls, the expression of Gallerimycin and Galiomicin was significantly upregulated 9.5-fold and 3.4-fold, respectively (Figure 7B). However, in larvae inoculated with C12 and after PSMPs, the expression of Gallerimycin was down-regulated 1.2-fold compared with C12 only, in contrast to the Galiomicin gene, which resulted in being down-regulated 3.2-fold compared with C12 only and 0.8-fold compared to the control. Again, compared to larvae injected with the C12 alone, when PSMPs are inoculated first, and after 2 h, infected with *Candida*, both peptides are down-regulated, indicating a lowering of the immune response and, therefore, a greater predisposition to infection, which is also corroborated by the greater mortality of the larvae (Figure 7B). Interestingly, in larvae inoculated at the same time with PSMPs and C12, the expression of both Gallerimycin and Galiomicin genes resulted in being down-regulated compared with C12 8.5- and 2.0-fold, respectively, showing a negative trend with respect to the other two conditions, suggesting that the co-exposure can inhibit the expression of antifungal peptides (Figure 7B).

### 2.9. Effects of PSMPs on the Gene Expression of HWP1 and ALS3

**Figure 8:**
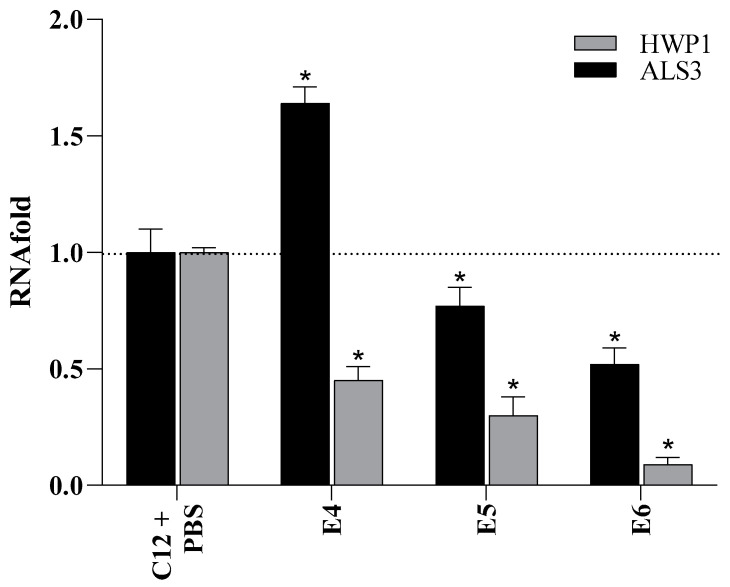
Gene expression analysis for mRNA of *C. albicans* virulence genes (*HWP1* and *ALS3*) in *G. mellonella* larvae. The figure shows larvae infected with C12 only, infected with C12, and exposed after 2 h to PSMPs (E4); those that underwent treatment with PSMPs and then C12 (E5); and, finally, samples treated simultaneously with PSMPs and C12 (E6). Relative mRNA expression levels measured using real-time PCR analysis and calculated via the 2^(−∆∆C(T))^ method. Actin gene was used as the housekeeping gene for the normalization of gene expression. Each sample was tested and run in duplicate. In this analysis, no-template controls were included. Error bars represent the SEM. * = *p* < 0.05 with C12 + PBS (Wilcoxon two-group test).

To test the hypothesis that PSMPs impact the infection ability of *C. albicans* in vivo, we investigated the expression of two genes known to be important in biofilm formation: *HWP1* (the hyphal cell wall protein) and *ALS3* (the agglutinin-like protein). At 24 h post-inoculation, in all three conditions tested, the levels of gene expression of *HWP1* and *ALS3* were significantly reduced, at least by 0.2-fold, except for *ALS3*, when PSMPs were administered after infection, as it increased by 1.6-fold compared to control (Figure 8).

## 3. Discussion

Plastic is a material found in packaging and products such as pharmaceuticals and cosmetics, textiles, face masks, and surgical instruments thanks to their properties, such as high durability, waterproofness, cost-effectiveness, and manufacturability with low energy requirements. Despite these benefits, plastics could persist in the environment as microplastics or nanoplastics, causing deleterious effects on human health. It is hypothesized that humans could ingest microparticles from everyday products, food, biomedical products, food containers, and drinking water, was well as by inhaling contaminated indoor and outdoor air or through cutaneous exposure through dust, clothing, and personal care items [20,21,22].

There is a growing concern regarding the potential effects that MPs have on human health. These particles can be ingested, inhaled, or adsorbed through the skin, key routes for their intracellular accumulation [23,24,25].

Evidence for the presence of pathogenic bacteria or fungi on plastics wastes is currently the focus of microplastics research. Polystyrene is widely used by consumers in a variety of products—for instance, in food containers—and ingestion is one of the primary exposure routes of PSMPs [26]. Pathogenic microorganisms can attach to the surfaces of PSMPs to form complex biofilms, in which they are protected from the immune system and antibiotics, leading to an inflammation condition [27].

*Candida albicans* is one of the most common pathogenic yeasts that can cause systemic infections in immunocompromised patients and hospitalized individuals both for the use of invasive devices and for the presence of some comorbidities, such as diabetes [28].

This study is the first aimed at investigating the potential interactive effects of environmentally relevant microplastics and fungal exposures.

Considering the potential toxic effects of PSMPs on cells, we first evaluated their effects on the viability of HT29 cells and found that 1 μm PSMPs were not significantly toxic up to relatively high concentrations. A FACS analysis on HT29 exposed to PSMPs for 24 h ascertained the internalization of PSMPs in accordance with other authors, who reported that only MPs ≤ 20 μm can penetrate tissues and cells, while those of 0.1–10 μm can also penetrate the cell membrane, blood–brain barrier, and placental barrier [29,30].

We further evaluated the effect of PSMPs on the infection ability of a clinical isolate of *C. albicans* both in vitro and in vivo. Our in vitro results showed that, when cells were exposed to PSMPs for 24 h and after were infected with C12 (E1 condition), the infection ability of fungal cells increased significantly. Epidemiological studies have shown that microplastic exposure may reduce body functions and contribute to an increased risk of infection, mainly due to a minor immunological defense [31]. For instance, Wang et al. [32], in a study, demonstrated that microplastics can promote influenza A virus infection by affecting endocytosis and the innate antiviral immune system. Particularly in E1, when we pretreated the cells for 24 h with PSMPs, *Candida* showed a greater invasive capacity, causing, at the same time, greater cellular damage, as confirmed by the increased IL-8 production and decreased IL-5 and G-CFS production. These results demonstrate that the PSMPs allowed fungi to colonize the gastric mucosal epithelial cells more quickly. For the in vivo study, we used the *G. mellonella* larvae model not only for its ease of use but also because the larval immune system exhibits remarkable structural and functional similarities to the innate immune response of mammals. Prior to the study of the effects of microplastics on the development of candidiasis in *G. mellonella*, we evaluated the susceptibility of larvae to PSMPs, confirming the non-toxicity at the tested concentrations. We also observed that the strain did not cause the death of the larvae in concentrations up to 10^5^ cells/larvae until 48 h, demonstrating low pathogenicity in the *G. mellonella* model. On the contrary, in the presence of PSMPs before or in conjunction with *Candida* infection, 80–100% mortality was evident after 48 h. The capacity of this strain, in all three conditions studied, to stimulate the immune system of *G. mellonella* showed that the PSMPs’ pre-exposure before *Candida* infection and the co-exposure may affect the immune system of larvae, thus promoting fungal infection. Furthermore, *HWP1* binded to *ALS3* mediates the adherence of *C. albicans* hyphae to each other [33], with both genes being relevant for biofilm formation. Here, only *ALS3* is overexpressed when larvae are infected after exposure, whereas *HWP1* results in being down-regulated. In the other two conditions (pre-exposure and co-exposure PSMPs + C12, PSMPs, and C12), both the selected genes were down-regulated with a decreasing trend. Since *HWP1* and *ALS3* participate in the early stages of biofilm formation in *C. albicans* [34], their down-regulation could be linked to the fact that larvae samples were collected when probably biofilm expansion is ending with the dispersion of planktonic cells. Our results, taken together, evidence that the accumulation of microplastics could have a dangerous health effect on organisms that ingest them not only due to their direct toxicity but also because they could facilitate the emergence of infectious disease by favoring the exposure of pathogens to humans.

In conclusion, even if microplastics are considered to be a novel environmental pollutant detected in the human body that is able to affect the metabolic level of organisms and cause various inflammatory reactions, this is the first report on the effects of microplastics on organisms infected with a pathogenic yeast, revealing its higher ability in promoting infection in vivo and in vitro. More studies are required to better understand the potential impact of polystyrene microplastics’ health risks on people infected by fungal pathogens.

## 4. Materials and Methods

### 4.1. Polystyrene Microplastics Characterization

Pristine polystyrene microplastics (PSMPs) of 1 µm diameter (5% *w*/*v* in deionized water) were purchased from “Micro Particles GmbH” (PS-Research 9003-53-6, according to Regulation (EC) No. 1907/2006; last updated on 6 December 2021; Berlin, Germany). Samples were loaded on an Isopore membrane filter, 0.4-micron pore size, and mounted on ultra-pure aluminum pin stubs. After platinum sputter coating with Leica EM ACE200, samples were analyzed using a Jeol field emission scanning electron microscope, JSM 6700-F.

The PSMPs stock solution was prepared at a concentration of 0.1 mg mL^−1^ in distilled water and properly diluted in different media for the subsequent use in experiments.

### 4.2. Cell and Fungal Culture

The HT29 epithelial cell line, derived from a colorectal adenocarcinoma, was maintained in Dulbecco’s modified Eagle’s medium with high glucose (DMEM; Sigma Aldrich Co., St. Louis, MO, USA), supplemented with 10% *w/v* fetal bovine serum (FBS; Sigma Aldrich Co., St. Louis, MO, USA), 2 mM L-glutamine (Sigma Aldrich Co., St. Louis, MO, USA), and 1% *w/v* penicillin–streptomycin (Sigma Aldrich Co., St. Louis, MO, USA).

Cells were kept in a humidified atmosphere of 5% CO_2_ at 37 °C and sub-cultured once a week according to the desired cell density. For the use, cells were enzymatically detached with trypsin/EDTA solution 0.25% (Sigma Aldrich Co., St. Louis, MO, USA) and counted using an inverted microscope. The HT29 cell line was kindly provided by Prof. Rosanna Capparelli (University Federico II, Naples, Italy).

The *C. albicans* (C12) that was isolated from feces and belonged to our laboratory collection was first identified by culturing colonies and, after, molecularly identified at ≥98% and compared with a reference strain, *C. albicans* JRP61, using nucleotide blast at GenBank (https://www.ncbi.nlm.nih.gov/genbank/, accessed on 9 January 2023). In this study the *C. albicans* ATCC 90028 was used too. C12 has also been previously characterized for its ability to form biofilm, for the different expressions of some virulence genes and for the different antifungal resistance showing to be susceptible to Amphotericin B, resistant to itraconazole and ketoconazole, and intermediate to fluconazole [35]. Strains were stored in 15% glycerol frozen at −80 °C and routinely maintained on Tryptone soya agar (TSA) (OXOID, Basingstoke, UK) with 1% glucose (VWR Chemicals, Radnor, Pennsylvania, United States of America). Liquid planktonic cultures were grown in Tryptone Soya Broth (TSB) (VWR chemicals, Leuven, Belgium) with 1% *v*/*v* glucose for 24 h at 37 °C, rotating at 200 rpm. For use, *Candida* cultures were standardized to 1 × 10^7^ cells mL^−1^.

### 4.3. PSMPs Cytotoxicity Assessment

Acute potential PSMPs cytotoxic effects on HT29 cells were evaluated to select suitable concentrations for the subsequent exposure experiment in both conditions in vitro and in vivo. To this end, 2 × 10^4^ cells/well were seeded in 96-well plates and incubated for 24 h at 37 °C and 5% CO_2_, and then they were treated with PSMPs at concentrations ranging from 0.1 to 100 μg mL^−1^. Untreated cells were used as the negative control. After the exposure time, MTT (Sigma Aldrich, St. Louis, MO, USA) solution was added to each well for 4 h at 37 °C. DMSO was added to dissolve the formazan crystals that were determined by measuring the absorbance at 570 nm, using a multiplate reader (SYNERGYH4 BioTek, Inc., Winooski, VT, USA). To calculate cell viability percentages, each treatment was compared to unexposed controls.

### 4.4. Citofluorimetric Assay

HT29 cells were exposed to 20 μg mL^−1^ of PSMPs for 3, 6, and 24 h. After exposure, cells were harvested, washed three times with PBS, and 1 μg mL^−1^ of the final concentration of propidium iodide (PI) (Sigma Aldrich, St. Louis, USA) was added at room temperature in the dark for 30 min. Cell fluorescence in each replicate was measured with a cytometer FACS can (BD Accuri™ C6 Flow Cytometer Biosciences, Piscataway, NJ, USA). Cells incubated with PI without PSMPs were the negative control.

### 4.5. Biofilm Formation Ability in Presence of PSMPs

The ability to form biofilm of C12 in the presence of PSMPs was evaluated by analyzing different steps of biofilm formation and development with three tests performed in vitro that consent to evaluate the initial attachment, formation, and maturation, as previously described [15]. PSMPs at range concentration of 10, 20, and 50 μg mL^−1^ were added to each well containing *Candida* cells at concentration of 0.5 × 10^5^ cells mL^−1^. The plates were incubated for 3 h, 6 h, and 24 h at 37 °C. After incubation, the plates were emptied and washed three times with PBS to remove non-adherent cells, and the total biofilm biomass was evaluated using the crystal violet (CV) staining methodology [36,37]. The percentage of cell adhesion was evaluated by measuring the absorbance at 570 nm, using a microtiter plate reader (SYNERGY H4 BioTek). The results were expressed as the mean of three independent experiments.

### 4.6. Candida Internalization and PSMPs Exposure

For screening the fungal strains’ infection ability, cells monolayers were infected with the two different *Candida* strains at a MOI (multiplicity of infection) of 1:1; 1:5, and 5:1 for 3 and 6 h [38] at 37 °C. After this time, cell monolayers were washed with PBS and treated with 5 µg mL^−1^ of Amphotericin B (Alfa Aesar by Thermo Fisher Scientific, Kandel, Germany) in DMEM (Sigma Aldrich Co., St. Louis, MO, USA) for 1 h. After incubation, cells were washed and lysed with a scraper, suspended in 1 mL of fresh PBS, and plated on Rose Bengal Agar (RBA) (VWR Chemicals, Leuven, Belgium). The results were expressed as the mean ± standard deviation (SD) of the number of intracellular *Candida*, as reported in Supplementary Figure S2, and expressed in Log_10_ CFU mL^−1^. Considering the same behavior of the two strains, all subsequent experiments were conducted only on *Candida* clinical isolate C12.

In the experiments of exposure of HT29 to PSMPs, the HT29 cells were infected with C12 always at MOI 1:5 for a total of 6 h and exposed to PSMPs at a concentration of 20 µg mL^−1^, following three different conditions: (E1) PSMPs for 24 h and then infection with C12 for further 6 h (pre-exposure); (E2) infection with C12 for 3 h and then PSMPs for further 3 h (post-exposure); (E3) PSMPs and C12 simultaneously for 6 h (co-exposure). For each condition of exposure to PSMPs (E1, E2, and E3), *Candida* internalization was evaluated. The amount of internalized fungal cells was enumerated as described above, and the results were expressed in Log_10_ CFU mL^−1^. The efficiency of internalization was calculated with the formula % efficiency of internalization = number of internalized *Candida*/total number of *Candida* × 100. Samples containing fungal cells alone were used as controls. For each condition (E1, E2, and E3), cellular damage was evaluated by determining the amount of lactate dehydrogenase (LDH) released into the medium, following the manufacturers’ instruction, using an LDH Cytotoxicity Assay Kit (Sigma Aldrich, Co., St. Louis, USA). Samples containing only epithelial cells, fungal cells, or PSMPs alone were used as controls.

### 4.7. Bio-Plex Assay for Cytokine Measurement

To investigate the effect of PSMPs and *C. albicans* on the HT29 cell immune response, pro-inflammatory cytokines were measured on the samples of HT29 cells infected with C12 and exposed to PSMPs according to the conditions (E1, E2, and E3) described above by performing the bio-plex assay. Briefly, HT29 cells were seeded in a 24-well plate and incubated at 37 °C in a 5% CO_2_ atmosphere, overnight. The next day, cells were washed using phosphate buffer saline (PBS, OXOID Limited Basingstoke Hampshire, England) and stimulated following the conditions E1, E2, and E3. Untreated cells were used as the control. After stimulation, cell medium was collected, centrifugated at 10,000× *g* at 4 °C for 10 min to remove cell debris, and stored at −20 °C until the analysis. A bio-plex pro human cytokine kit (Bio-Rad, Hercules, CA, USA, catalog #M5000031YV) was used to determine the IL-5, IL-8, TNF-α and G-CSF release in cell supernatant, according to the manufacturer’s instructions [39].

### 4.8. Monitoring G. mellonella Larvae

To establish whether PSMPs cooperate with the *C. albicans* isolate to promote infection and immune response in vivo, *G. mellonella* larvae were used. First, we assessed the PSMPs toxicity and the pathogenicity of C12 with a larval killing assay, as previously described [40]. Briefly, 20 *G. mellonella* larvae for each group, with a body weight between 200 and 300 mg, were injected directly into the hemocoel right proleg region, using a 50 µL Hamilton syringe with a 26 g needle. Larval killing assays were carried out at 37 °C, using different inocula of 10^5^, 10^6^, and 10^7^ yeast cells/larva or 10, 20, 50, and 100 µg mL^−1^ of PSMPs, and death was monitored daily over 3 days by making a visual inspection of the color and lack of movement after stimulation. Then, the same three different conditions tested in vitro were also carried out in vivo. Larvae were infected with 10^5^ *Candida* cells/larvae (concentration previously determined) at the last right proleg, and after 2 h, they were treated with PSMPs by injecting 20 µg mL^−1^ into the last left proleg (volume of 10 μL), and, vice versa, they were pretreated with PSMPs and then injected with *Candida* cells (E4 and E5, respectively). The synergy between microplastics and yeast was assessed by administering both the yeast suspension and PSMPs in a single injection (E6). Larvae were incubated at 37 °C and monitored daily for survival. Groups of larvae untreated with the yeast or microplastics served as a blank control. The experiments were performed in triplicate. Three live larvae from each experimental group after 24 h of treatment were snap-frozen in liquid nitrogen and kept for subsequent experiments.

### 4.9. Analysis of Gallerimycin and Galiomicin Gene Expression

RNA was extracted at 24 h post-treatment. The three live larvae from each experimental group snap-frozen in liquid nitrogen were ground to a powder by mortar and pestle in TRIzol (Invitrogen, Paisley, UK). The samples were further homogenized using a TissueLyser II (Qiagen, Valencia, CA, USA) and steal beads of 5 mm diameter (Qiagen, Valencia, CA, USA). RNA was extracted with an RNeasy minikit (Qiagen, Valencia, CA, USA), following the manufacturer’s protocol. The quality and amount of purified RNA were analyzed spectrophotometrically with Nanodrop2000 (Thermo Scientific Inc., Waltham, MA, USA). A total of 1000 ng of RNA was reverse transcribed with the QuantiTect Reverse Transcription Kit (Qiagen, Valencia, CA, USA), used as described by the manufacturer. Afterwards, Real-Time PCR was performed using the QuantiTect SYBR Green PCR Kit (Qiagen, Valencia, CA, USA) in a final volume of 25 μL, with 100 ng of cDNA, 1 μM of each primer, and 12.5 μL of QuantiFast SYBR Green PCR Master Mix (2×). The PCR cycling profile consisted of a cycle at 95 °C for 5 min; and 40 two-step cycles at 95 °C for 15 s, at 60 °C for 60 s. Quantitative RT-PCR analysis was conducted using the 2^(−∆∆C(T))^ method [41].

RT-PCR was performed in a Rotor-Gene Q cycler (Qiagen, Valencia, CA, USA). All primers used for quantitative PCR (qPCR) studies are shown in Supplementary Table S1. At the end of each test, a melting curve analysis was performed (plate read every 0.5 °C from 55 to 95 °C) to determine the formation of the specific products. Each sample was tested and run in duplicate. No-template controls were included. mRNA levels in the different treatments were compared by ANCOVA (analysis of covariance). The control and the treatment groups in various assays were compared and analyzed using a Wilcoxon two-group test, and data with *p*-values < 0.05 were considered statistically significant [42]. Transcriptional activation is represented by the RNA fold change in the expression; for the galiomicin and gallerimycin genes evaluation in *G. mellonella*, actin was used as the housekeeping gene, and for *HWP1* and *ALS3* genes in *C. albicans*, actin was used as the housekeeping gene.

### 4.10. Statistical Analysis

For statistical analysis, GraphPad Prism software (8.02 for Windows, GraphPad Prism Software, La Jolla, CA, USA, www.graphpad.com, accessed on 7 September 2023) was used. Data were obtained from two or three different experiments and expressed as the mean ± standard deviation (SD) or the Standard Error of the Mean (SEM). A one-way analysis of variance (ANOVA), followed by Dunnett’s or Tukey’s multiple comparation test, was used to compare the treated to control groups. Survival curves were plotted using the Kaplan–Meier method and the log-rank (Mantel–Cox) test. To evaluate the expression gene, REST software (Relative Expression Software Tool, Weihenstephan, Germany, version1.9.12) was used, and the Wilcoxon two-group test was used to evaluate the difference with the control.

## Data Availability

Data are contained within the article and supplementary materials.

## Supplementary Materials

The following supporting information can be downloaded at https://www.mdpi.com/article/10.3390/ijms25010012/s1.
