# Supplementary material for: Polystyrene Microplastics Exacerbate Candida albicans Infection Ability In Vitro and In Vivo
Source: Int J Mol Sci. 2023 Dec 19;25(1):12. doi: 10.3390/ijms25010012 (PMC10778850; doi:10.3390/ijms25010012)
Supplement: Supplementary file 1 [file ijms-25-00012-s001.zip › ijms-2731314-supplementary.pdf]

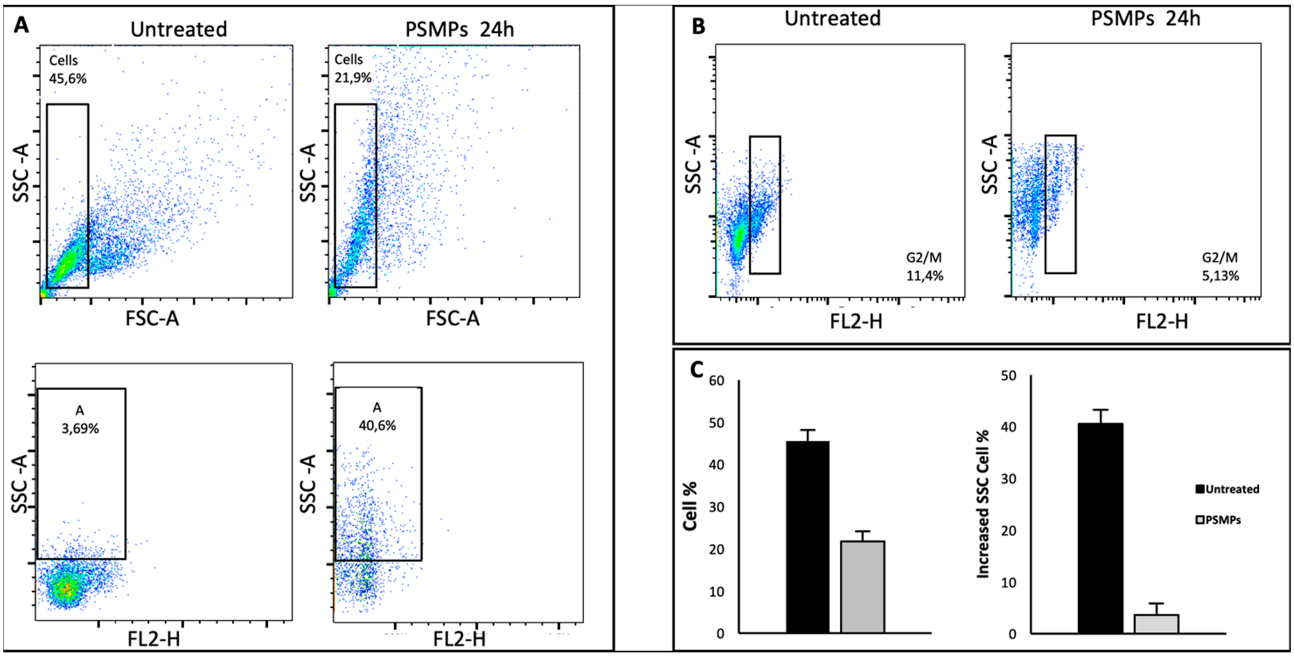


**Figure S1.** Cytofluorimetric Cell morphology analysis. HT29 cells were exposed to 20 µg mL$^{-1}$ of PSMPs for 24 h. A) The representative dot plots for treated and untreated cells are shown in linear FL2-H; B) and in logarithmic FL2-H; C) Histograms represent the mean ± standard deviation of two experiments made in triplicate of percentage of cells in the untreated and PSPMPs treated groups (left), and the percentage of cells with increased SSC (right).

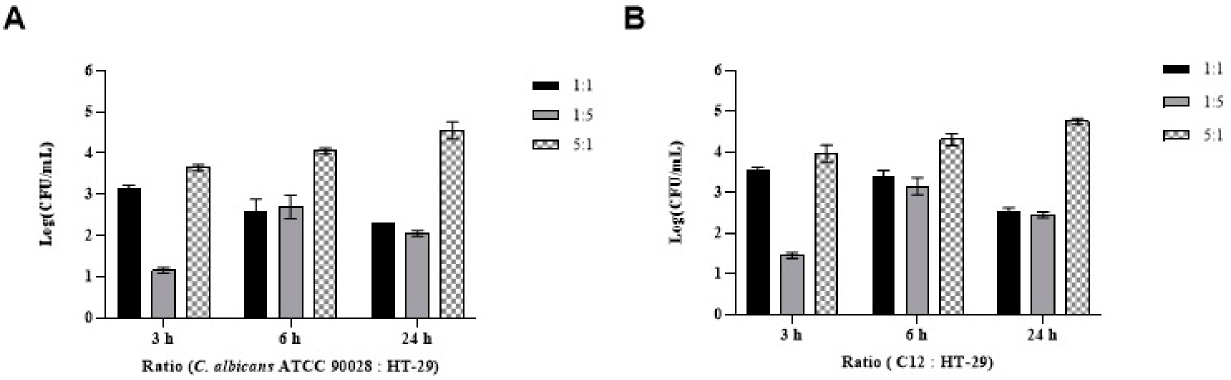


**Figure S2.** Effect of *C. albicans* ATCC 90028 and C12 clinical isolate at MOI 1:1, 1:5 and 5:1 at different time on HT29 cells.

**Table S1.** Gene-specific primers used for real-time RT-PCR.

| Primer Name | Primer Sequence (5′-3′) | Amplicon Length (bp) |
|---|---|---|
| *G.mellonella_actin_F* | GGACTTGTACGCCAACACAG | 196 |
| *G.mellonella_actin_R* | CCACATCTGCTGGAATGTCG | |
| *G.mellonella_galiomycin_F* | GGTGCGACGAATTACACCTC | 101 |
| *G.mellonella_galiomycin_R* | TCGCACCAACAATTGACGTT | |

| | | |
|---|---|---|
| ***G.mellonella_gallerimycin*_F** | GAAGATCGCTTTCATAGTCGC | 173 |
| ***G.mellonella_gallerimycin*_R** | TACTCCTGCAGTTAGCAATGC | |
| ***C.albicans_actin*_F** | AGCCCAATCCAAAAGAGGTATT | 153 |
| ***C.albicans_actin*_R** | GCTTCGGTCAACAAAACTGG | |
| ***C.albicans_HWP1*_F** | CAGCCACTGAAACACCAACT | 135 |
| ***C.albicans_HWP1*_R** | CAGAAGTAACAACAACAACACCAG | |
| ***C.albicans_ALS3*_F** | CTAATGCTGCTACGTATAATT | 201 |
| ***C.albicans_ALS3*_R** | CCTGAAATTGACATGTAGCA | |
